# The simultaneous occurrence of lichen planopilaris and alopecia areata: A report of two cases and literature review

**DOI:** 10.1002/ccr3.2963

**Published:** 2020-05-24

**Authors:** Fatemeh Mohaghegh, Bahareh Bahrami, Mina Saber

**Affiliations:** ^1^ Department of Dermatology Skin Diseases and Leishmaniasis Research Center School of Medicine Isfahan University of Medical Sciences Isfahan Iran

**Keywords:** alopecia, alopecia areata, lichen planopilaris

## Abstract

Although the coexistence of alopecia areata and lichen planopilaris is rare, if alopecic patches appear abruptly, this possible association should be kept in mind.

## Graphical abstract

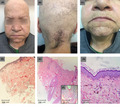

## 1 INTRODUCTION

Lichen planopilaris (LPP) is the most common form of scarring alopecia that is considered a trichologic emergency by some experts. Although its precise pathogenesis is unknown to date, some researchers have suggested the role of immune dysregulation in its incidence. Common clinical manifestations of LPP include single or multiple patches of alopecia in the vertex or parietal region_._ ^1^, ^2^

Alopecia areata (AA) is also a common cause of noncicatricial alopecia that could occur as patchy or diffuse hair loss with partial or complete recovery. It has been often considered a T cell–mediated disease with the loss of immune privilege in the hair follicle.^3^

Lichen planopilaris is typically considered an irreversible process, unlike AA, which is frequently associated with partial or complete recovery. The concomitant association of these two conditions is a rare phenomenon.^4^

This article reviews the existing literature on the subject and describes two cases of the coexistence of LPP as the prototype of scarring alopecia and AA as a nonscarring alopecia.

## 2 CASE REPORTS

### 2.1 Case 1

**Figure 1:**
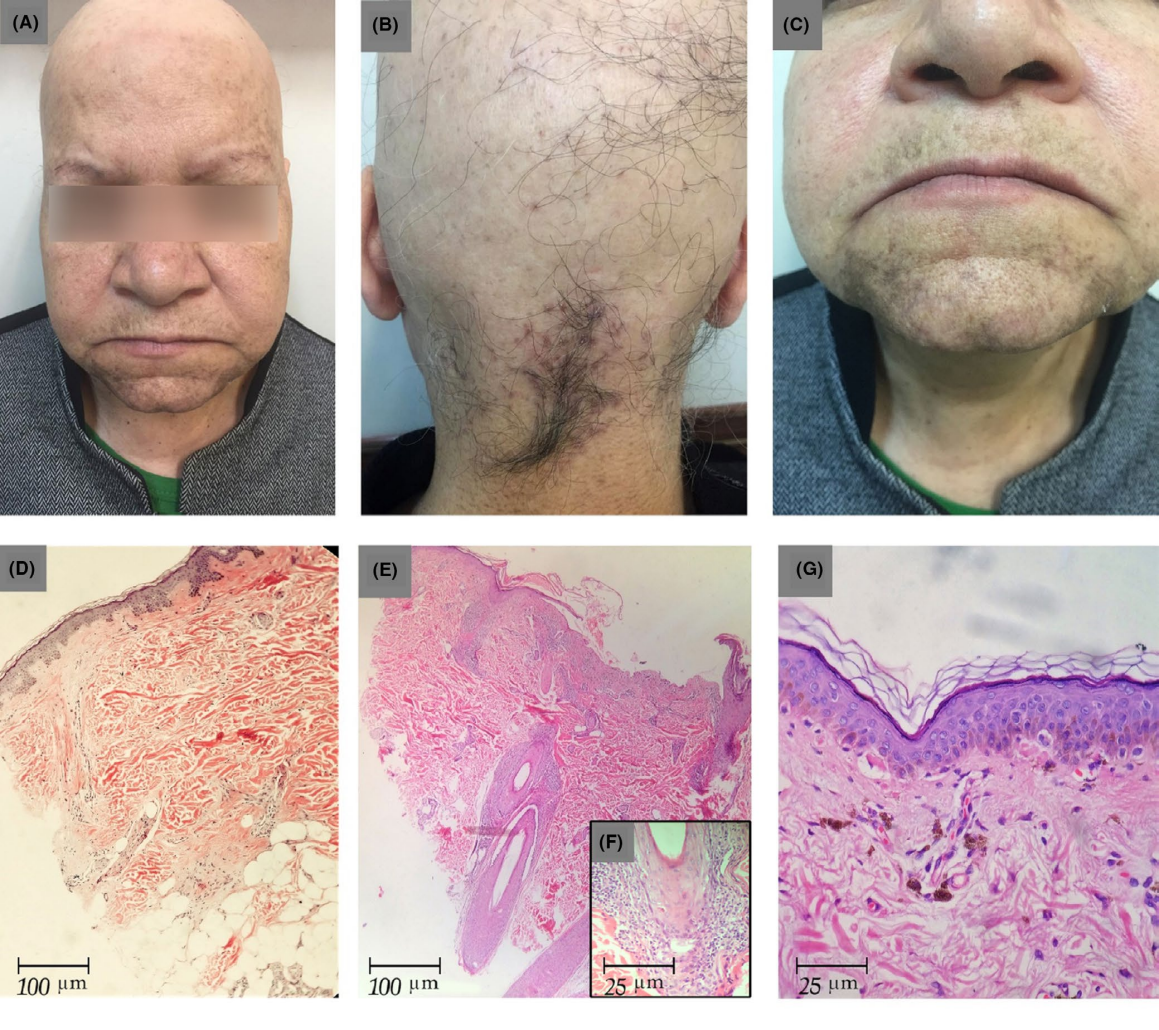
Clinical and histopathological findings. A, Diffuse hair loss of the scalp and partial hair loss with perifollicular erythema and scaling of the eyebrow. B, Perifollicular erythema and scaling in some residual terminal hair of occipital area. C, Gray‐brown macules and patches on the chin and top of the lip. D, Complete loss of follicles. A fibrous streamer extends along the site of the previous follicle (HE, ×100). E, F, The lichenoid infiltrate confined to perifollicular location (HE, ×100). The insert shows basal cell vacuolization and civatte bodies (HE, ×400). G, There is vacuolar degeneration of basal layer with subtle lymphocytic infiltration and marked melanin incontinence (HE, ×400)

A 64‐year‐old woman, otherwise healthy, who was a known case of alopecia areata totalis, referred to a dermatology clinic due to progressive eyebrow hair loss and skin pigmentation of the face for 6 months. She noted the sudden onset of asymptomatic diffuse hair loss 20 years back that had progressed to the entire scalp. Some residual hair remained in her occipital scalp (Figure 1A‐C).

During these 20 years, the patient was treated with topical and systemic corticosteroids, but her condition had not improved.

Six months ago, she had also noticed asymptomatic gray‐brown macules and patches in the chin and top of the lip. Over these 6 months, the lesions had increased in number and had spread to other parts of the face skin and scalp. She did not have any preceding erythema before the appearance of hyperpigmentation. These lesions were accompanied by hair loss in the eyebrow. Some residual eyebrow and scalp hair showed perifollicular erythema and scale on the physical examination. No nail and mucosal involvement was discerned. The systemic examination results were within the normal limits.

Her laboratory studies, including complete blood count, metabolic profile, lipid profile, serum vitamin D level, thyroid hormone level, and urinalysis, were within the normal limits. The biopsy of the hairless region of her scalp showed complete loss of the hair follicles accompanied by fibrous streamers extending along the site of the previous follicles, suggesting a diagnosis of AA (Figure 1D). Another biopsy of the residual occipital scalp hair showed decreased hair density and lichenoid inflammation in the peri‐isthmus region, consistent with LPP (Figure 1E‐F).

In addition, the skin biopsy of the pigmented macules in the chin revealed epidermal atrophy and vacuolar degeneration of the epidermal basal layer. The lymphocytic inflammatory infiltrate was subtle, but melanin incontinence was strongly expressed (Figure 1G). Given these clinical and pathological findings, the patient was diagnosed with lichen planus pigmentosus.

She was given topical mometasone and pimecrolimus; however, she did not show for the follow‐up.

### 2.2 Case 2

A 29‐year‐old male patient presented with the chief complaints of asymptomatic patchy hair loss in the scalp and beard for the past 2 years. He was a known case of LPP from 12 years ago and had been treated with prednisolone and mycophenolate mofetil for 3 years. His medications were discontinued after clinical improvement, and hair transplantation was performed for him. Eight years after the hair transplantation, he came back with asymptomatic well‐defined patches of nonscarring alopecia in his temporal region of the head and beard.

**Figure 2:**
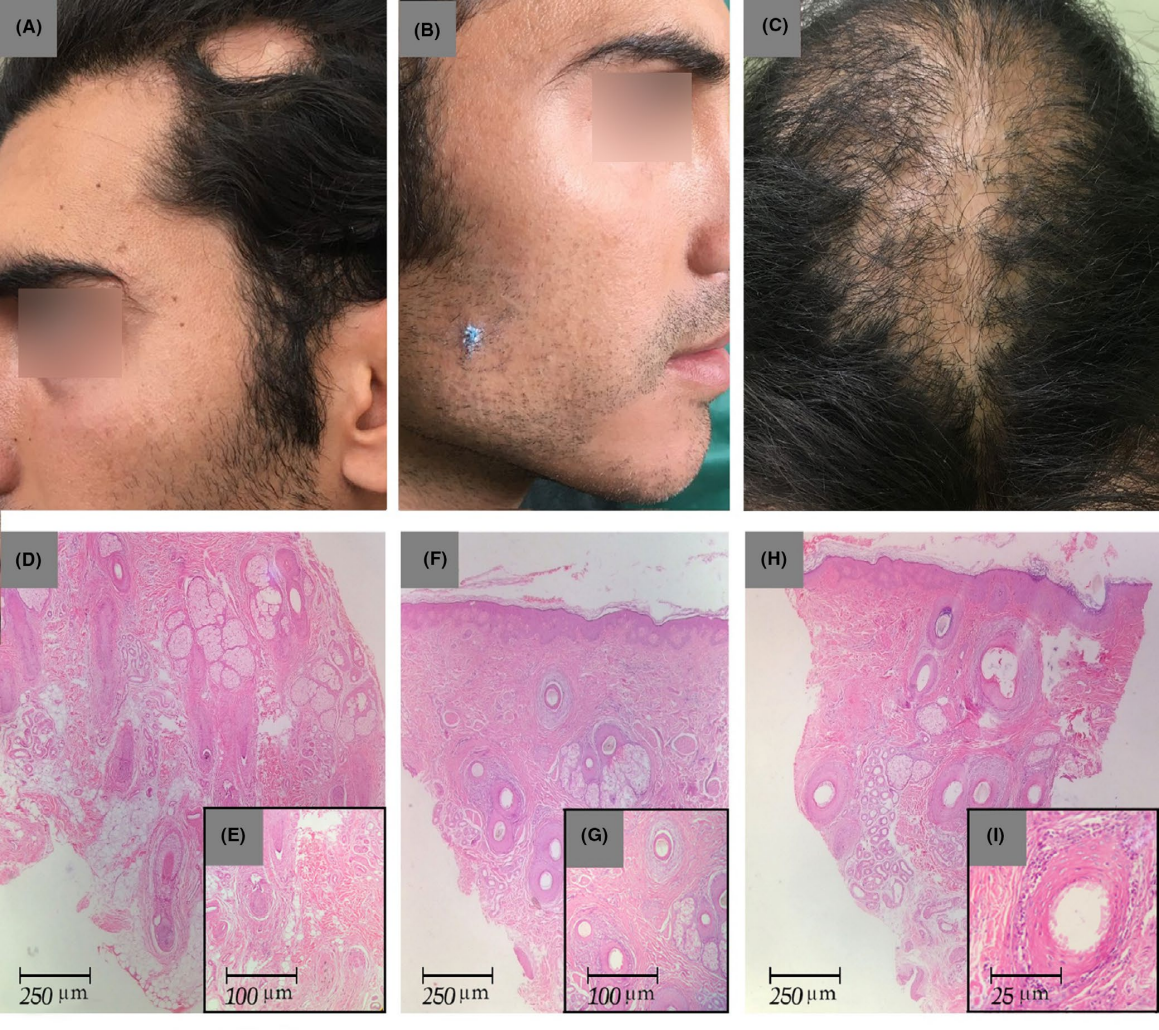
Clinical and histopathological findings. (A) A well‐defined patch of nonscarring alopecia at the temporal region of scalp. B, Irregular patch of scarring alopecia with perifollicular erythema and a patch of nonscarring alopecia in beard area. C, Diffuse scaring alopecia on the top of the head and vertex. D, E Vertical section showing a decreased number of terminal anagen hairs and increased number of telogen and vellus‐like follicles. A few peribulbar lymphocytic infiltration around miniaturized hairs is seen (HE, ×40). The insert shows more details (HE, ×100). F, G, There is lichenoid infiltration around upper port of terminal follicles with fibromucinous change around another one (HE, ×40; HE, ×100, respectively). H, I, A subtle lichenoid infiltration and prominent fibromucinous change are around upper port of a terminal anagen follicle. The epidermis is normal (HE, ×40). The insert shows lichenoid infiltration involving the basal layer of the follicular epithelium (HE, ×400)

Upon examination, there was a well‐defined patch of nonscarring alopecia, size 4 × 4 cm, in the left temporal region of the scalp (Figure 2A). In addition, the areas of diffuse scarring alopecia were observed on the top of the head and vertex, where hair transplantation had been performed (Figure 2C). There were also small irregular patches of scarring alopecia with remarkable perifollicular erythema, and positive anagen pull test was accompanied by nonscarring alopecia, size 3 × 3 cm, in the beard (Figure 2B). He had neither pruritus nor burning sensations. Moreover, there was no hair loss in his eyebrows. Mucocutaneous and nail changes related to lichen planus were not present either. All the laboratory data were unremarkable.

**Figure 3:**
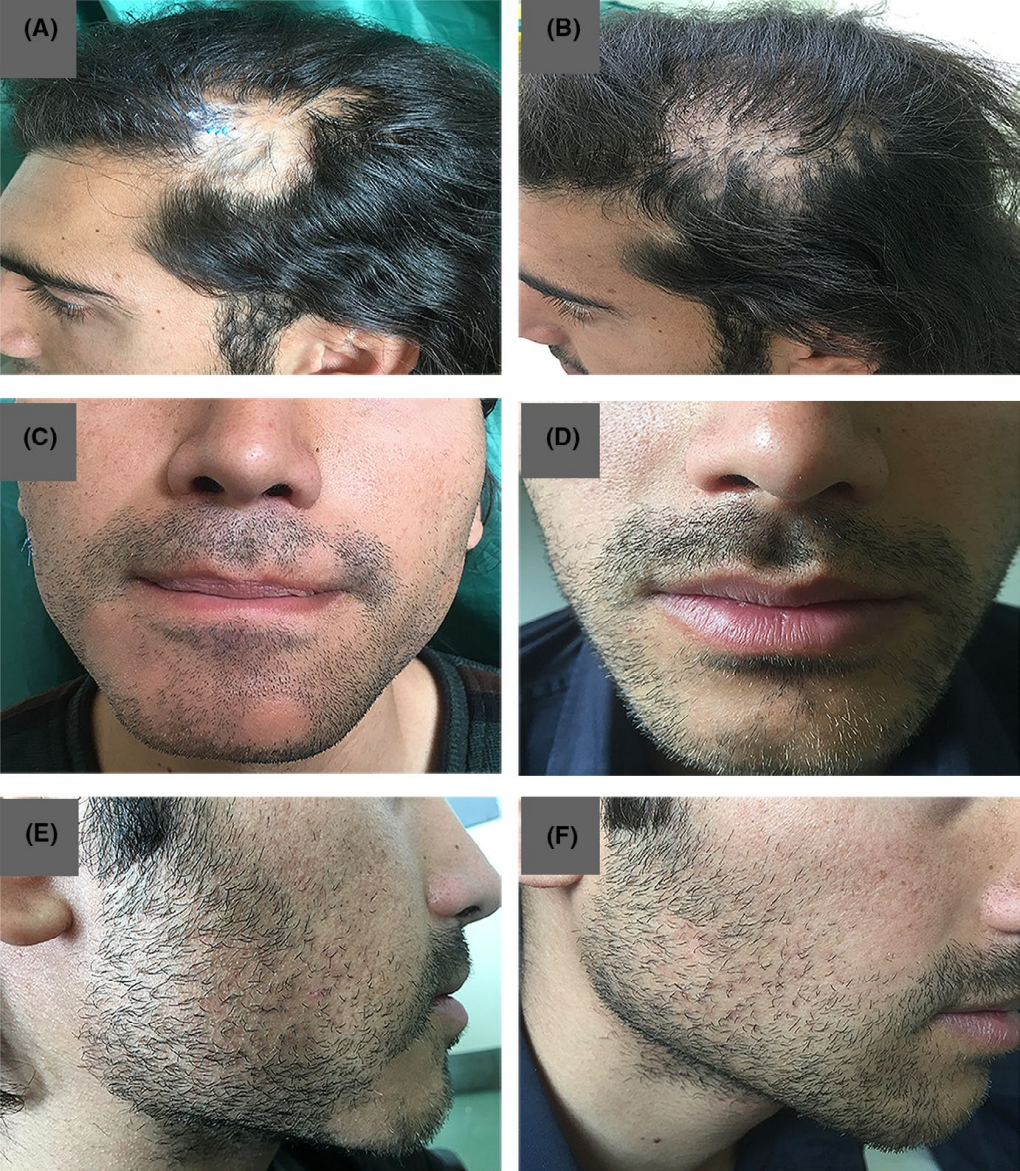
A, Temporal region, before treatment. C, E, Beard region before treatment. B, Temporal region, after a 3‐mo treatment. D, F, Beard region, after a 6‐mo treatment

The biopsy specimen of the temporal nonscarring alopecia patch showed a few vellus follicles in the mid‐dermis with sparse peribulbar lymphocytic infiltrate, as consistent with AA (Figure 2D‐E). Another biopsy of the beard region revealed lymphocytic infiltrate around the upper portion of the hair follicles and vacuolization of the basal layer of the epidermis with fibromucinous change, as consistent with LPP (Figure 2F‐G). The histopathological examination of a tissue taken from the vertex area confirmed the diagnosis of LPP (Figure 2H‐I). He started a combination therapy with intralesional triamcinolone in the AA lesions in combination with systemic prednisolone (200 mg/week) and methotrexate (15 mg weekly) for 3 months. The follow‐up visit after 3 months revealed a decrease in the patient's recorded inflammatory signs in the areas of the scalp and beard affected by LPP. The alopecia areata of his temporal region and beard had nearly resolved with white hair regrowth (Figure 3). Due to his poor compliance with the treatment, the patient stopped all the medications and only continued with the intermittent use of topical clobetasol.

## 3 DISCUSSION

The presented cases show an unusual coexistence of cicatricial and noncicatricial alopecias. Case 1 was a known case of severe alopecia areata who developed LPP in the residual hair in the scalp and eyebrow area. In contrast, case 2 was a known case of LPP who developed patchy alopecia areata in the scalp and beard area.

Several studies have formerly discussed the association between lichen planus (LP) and AA and proposed an autoimmune pathogenesis for both diseases. ^5^, ^6^, ^7^

There have also been reports on the colocalization of AA and LP.^8^ Although LPP is a follicular variant of LP, a similar relationship between LPP and AA has not been clearly demonstrated.

Athena et al found higher odds of AA development in patients with LPP compared to controls.^9^ There are few case records of the coexistence of AA in patients with LPP.^1^, ^10^

In a large study of 334 patients with LPP, none of the subjects had concurrent AA.^4^

It seems that patients with frontal fibrosing alopecia (FFA), as a variant of LPP, have a higher chance of developing AA than LPP patients. The association of FFA as a scarring alopecia with androgenetic alopecia as a nonscarring one has been reported before in the literature_._ ^11^

In a study by Banka et al^12^, 13% of patients with FFA had AA as well.

In another study, 6% of the FFA patients had simultaneous AA.^13^ In contrast, in a large series of FFA patients, only two had concomitant AA.^11^ Previous studies have thus demonstrated inconsistent results.

Nevertheless, the association of these separate disorders may be attributed to concurrent autoimmune diseases. In other words, the mechanism of this association is the loss of immune privilege (IP) in the hair follicles. Hair follicle is an immune‐privileged site that is normally protected by local immunosuppressive mechanisms.^14^ The failure of these mechanisms contributes to the pathogenesis of autoimmune hair loss disorders, including AA and LPP. Patients with any of these autoimmune hair disorders who have lost IP in one part of the hair follicle might be predisposed to IP loss at another site as well. As a result, LPP patients who have lost their IP at the bulge region may also be affected by AA, which is identified by the loss of IP at the bulb region, or vice versa.^15^

In this study, we present a case of AA that consequently developed LPP in the remaining hair. To the best of our knowledge, there have been no reports of severe AA with a subsequent development of LPP in the remaining hair. Most previous cases have shown concurrent LPP and AA in LPP patients who have also had AA as a comorbidity. ^1^, ^9^, ^10^

The reason may be that the bulge region has a stronger immune protection than that of the hair bulb. Patients with LPP who have lost their IP at well‐protected bulge regions are therefore more prone to IP loss at less‐protected bulb regions. The inverse scenario for AA patients is, however, less likely, which might explain why AA (prototype of bulb IP collapse) is more common than LPP (prototype of bulge IP collapse) in clinical practice.^14^

On other words, several mechanisms uphold hair follicle immune privilege such as downregulation of major histocompatibility complex (MHC), expression of nonclassical MHC class Ib, and local generation of immunosuppressants.^14^

Any of these mechanisms and cellular composition differ in the bulb (proximal part) vs bulge (distal part) of hair follicle. Indeed, immune privilege collapse in one region of hair follicle does not necessarily mean an increased risk of experiencing collective collapse.^16^ This hypothesis can somehow be used to describe why simultaneous occurrence is rare between LPP and AA. Also, we have a long way to discover and to address many intriguing questions about this issue.

As to treatment, since these two conditions are a kind of follicular autoimmune diseases, one stone kills two birds. Therapeutic modalities include corticosteroids (topical, intralesional, and systemic) and systemic immunomodulator medications such as methotrexate.^17^, ^18^

In summary, this article has reported on two cases of simultaneous scarring and nonscarring alopecia. These reports confirm the generally accepted knowledge that “Individuals with an existing autoimmune disorder are at an increased risk of a second disorder.” Making a diagnosis of AA and LPP is sometimes challenging, and the possible coexistence of these two conditions must be kept in mind.

## CONFLICT OF INTEREST

None.

## AUTHOR CONTRIBUTIONS

FM: evaluated the skin biopsies and contributed to the editing of the manuscript. BB: wrote and edited the manuscript. MS: followed up the patients and wrote and edited the manuscript.
